# Marginalized mites: Neglected vectors of neglected diseases

**DOI:** 10.1371/journal.pntd.0008297

**Published:** 2020-07-23

**Authors:** Thomas Weitzel, Benjamin L. Makepeace, Ivo Elliott, Kittipong Chaisiri, Allen L. Richards, Paul N. Newton

**Affiliations:** 1 Laboratorio Clínico, Clínica Alemana de Santiago, Facultad de Medicina Clínica Alemana, Universidad del Desarrollo, Santiago, Chile; 2 Instituto de Ciencias e Innovación en Medicina (ICIM), Facultad de Medicina Clínica Alemana, Universidad del Desarrollo, Santiago, Chile; 3 Institute of Infection & Global Health, University of Liverpool, Liverpool, United Kingdom; 4 Centre for Tropical Medicine and Global Health, Nuffield Department of Medicine, University of Oxford, Oxford, United Kingdom; 5 Lao-Oxford-Mahosot Hospital-Wellcome Trust Research Unit, Mahosot Hospital, Vientiane, Lao PDR; 6 Department of Helminthology, Faculty of Tropical Medicine, Mahidol University, Bangkok, Thailand; 7 Department of Preventive Medicine and Biostatistics, Uniformed Services University of the Health Sciences, Bethesda, Maryland, United States of America; Johns Hopkins Bloomberg School of Public Health, UNITED STATES

## Introduction

Trombiculid mites are tiny arthropods with a high species diversity and global distribution. The larvae (called “chiggers”) of some species are vectors of scrub typhus, a human febrile disease of potential lethality. Although scrub typhus threatens over a billion people within the Asia Pacific, its ecology is poorly understood compared to other vector-borne diseases. The recent discovery of scrub typhus in Chile and the Arabian Peninsula suggests a much wider distribution and highlights our profound knowledge gaps. How could scrub typhus be unrecognized outside Asia-Pacific region, where it has been known for more than 2,000 years? We hypothesize that the main reason is that scrub typhus is transmitted by mites, a vector group that has been overlooked by health organizations and scientists for decades. As a change of viewpoint, we suggest recognizing mites, these tiniest of all known arthropods transmitting infectious agents, as neglected vectors.

## The expanding spectrum of chigger-borne rickettsioses

Since 2006, an unexpected endemic rickettsiosis, scrub typhus, was detected in various regions in southern Chile [1–3]. This ancient Asian-Pacific disease was unknown in the New World, and its discovery in South America raised many questions, including origin, distribution, and routes of transmission [4]. Speculations included an association with recent worldwide phenomena of globalization and climate change or the migration of animals such as rodents and birds [4,5]. The change of paradigm was supported by the isolation of a new species, *Candidatus* Orientia chuto, from a patient visiting the Arabian Peninsula and by additional serological and molecular data from countries in Africa, suggesting a much broader distribution of scrub typhus outside the traditional tsutsugamushi triangle [6].

Since trombiculid mites, the vector and reservoir of scrub typhus in the Asia Pacific, had never been documented in the Chilean region where scrub typhus was initially discovered, other vectors such as terrestrial leeches were suspected [1]. However, a recent study demonstrated that trombiculid mites are endemic in southern Chile and that they are infected with the Chilean *Orientia* strain [7]. Interestingly, the infected mites belong to the genus *Herpetacarus*, which has never been associated with scrub typhus elsewhere. Together with phylogenetic analyses, showing that the isolate from Chile is distinct to other *Orientia* species [8], these findings suggest an ancient origin rather than recent introduction of the pathogen. Thus, generations of medical doctors and scientists have missed this important rickettsial disease. As scrub typhus is effectively and inexpensively treated with tetracyclines, such disregard has key public health implications. How was this possible?

## Chiggers: The neglected vector

The increasing awareness and research on vector-borne infections has led to tremendous advances in our knowledge of medically important arthropods. Similarly, the number of known rickettsial infections worldwide has more than tripled in the last three decades due to the use of new molecular diagnostic techniques [9]. How could this progress regarding arthropod-borne infections have spared scrub typhus, the rickettsial disease with the highest morbidity and mortality worldwide [10]?

**Fig 1:**
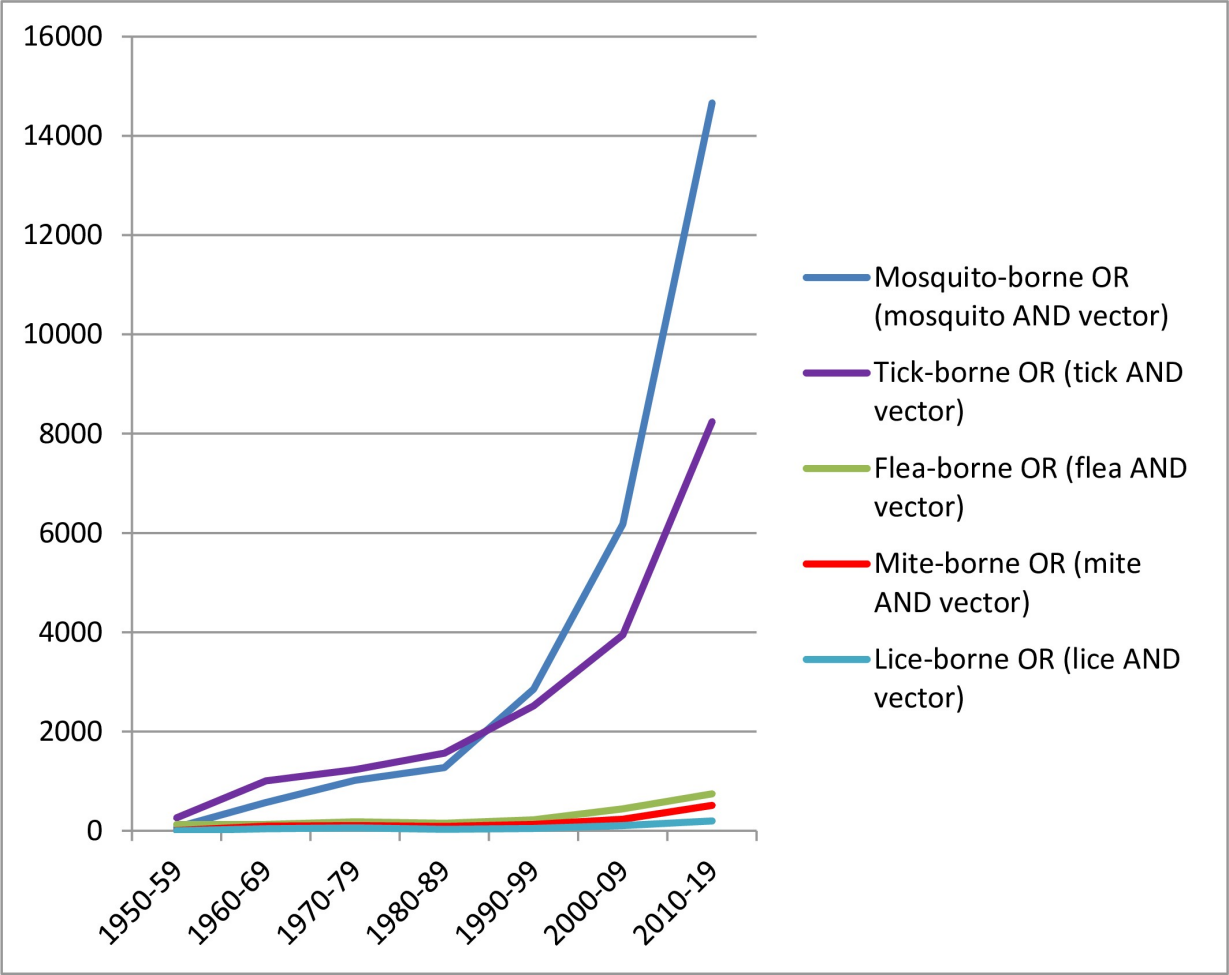
PubMed results for “all fields” searches regarding certain arthropods as vector. Numbers represent hits per decade (January 1, 1950 until December 31, 2019).

We hypothesize that the principal cause is that scrub typhus is transmitted by trombiculid mites, a vector group that has been overlooked by health organizations and scientists for decades. Reasons behind this incomprehension might lay in the traditional association of these vectors with the (rather exotic) tsutsugamushi triangle, combined with methodological challenges related to their secluded life cycle and the minute size of larvae. The negligence also affects other mite-borne neglected diseases, such as rickettsial pox [11]. Therefore, mites, these smallest of all known arthropods transmitting infectious agents, should be recognized as neglected vectors. To underline this viewpoint, we assessed scientific interest in different arthropod vectors using PubMed entries (https://www.ncbi.nlm.nih.gov/pubmed) as a proxy. This surrogate analysis revealed that since 1950, the number of publications grew exponentially but only for mosquitos and ticks (Fig 1). In sharp contrast, the interest in mites as vectors was low during the past decades. It was not always so; there were reactive paroxysms of mite research during the medical emergency of scrub typhus in the Asia Pacific amongst combatants during World War II [5], but, even though the numbers affected must now be much greater, impetus declined dramatically. Following the logic of our analysis, the body of knowledge regarding mites as vectors in 2019 is at the same surrogate level it was for ticks and mosquitos in the years 1968 and 1973, respectively (1,100 to 1,200 cumulative publications). A similar “All Fields” search showed that the relationship between mites as vectors and climate change has almost been ignored up to now (11 publications), in comparison to mosquitos (513 publications) and ticks (245 publications). If the search was limited to “Title/Abstract,” the term “chigger OR trombiculid OR *Leptotrombidium*” scored 596 hits, compared to 15,798, 13,368, and 6,983 for *Aedes*, *Anopheles*, and *Ixodes*, respectively. A recent systematic review of *Orientia* ecology, the first since 1974 [12], also highlighted the limited knowledge on trombiculid mites; furthermore, many of the included studies were difficult to access and/or were published in Chinese, Japanese, Korean, or Russian [5].

The scientific negligence of our understanding of mites as vectors seems pervasive. Although scrub typhus threatens more than 1 billion people worldwide [10], a recent “global brief on vector-borne diseases,” published by the World Health Organization (WHO), does not include mites as vectors [13]. Within the four conferences organized by the “European Network for Neglected Vectors and Vector-Borne Diseases (EurNegVec),” not a single presentation on mites can be found (https://www.eurnegvec.org/). Similarly, the main reviews on the impact of climate change on vector borne diseases published in 2019 do not mention trombiculid mites [14, 15], although climate is a key factor of their distribution [5].

## A call for more mite research

There are no dedicated funding schemes, rare presentations at tropical medicine conferences, and very few acarologists worldwide able to morphologically distinguish trombiculid and other mites to species level. These experts are crucial, since genetic or proteomic identification techniques, which are available for mosquitos and ticks, are in their infancy for mites. Recognition of this data chasm might help to motivate more researchers and funding organizations to dedicate time and money to these tiny but important vectors. Indeed, it is highly likely that apart from chiggers, the blood-feeding gamasid mites are also involved in the transmission of a variety of known and unknown zoonotic infections. Both trombiculid and gamasid mites harbor a variety of potential bacterial pathogens and have been strongly implicated in the epidemiology of Korean hemorrhagic fever (Hantaan virus) [16,17]. Furthermore, they may have a role to play in vectoring *Bartonella* spp., *Borrelia* spp., and *Rickettsia* spp. [18–20]. After decades of ignorance, there is an urgent need to catch up; trombiculid and other neglected mites might actually be more prolific and widespread vectors of zoonotic agents than is currently assumed.
